# Evaluation of the High- and Low-Temperature Performance of Asphalt Mortar Based on the DMA Method

**DOI:** 10.3390/ma15093341

**Published:** 2022-05-06

**Authors:** Yanzhu Wang, Xudong Wang, Zhimin Ma, Lingyan Shan, Chao Zhang

**Affiliations:** 1 School of Transportation Science and Engineering, Harbin Institute of Technology, Harbin 150090, China; wangyzh0102@163.com (Y.W.); 18b332002@stu.hit.edu.cn (C.Z.); 2 Research Institute of Highway, Ministry of Transport, Beijing 100088, China; mzm1755709496@163.com (Z.M.); ly.shan@rioh.cn (L.S.)

**Keywords:** asphalt mortar, dynamic mechanical analysis, phase transition, high- and low-temperature performance

## Abstract

Asphalt mortar is a typical temperature-sensitive material that plays a crucial role in the performance of asphalt mixture. This study evaluates the high- and low-temperature performance of asphalt mortar based on the dynamic mechanical analysis (DMA) method. Temperature-sweep tests of asphalt mortars were conducted using the DMA method under fixed strain level, frequency, and heating rate conditions. The dynamic mechanical response curves, characteristic temperature, and other indices were obtained and used to investigate the high- and low-temperature performance of asphalt mortar. The results showed that the phase transition temperatures T_1_, T_0_, and T_g_ can be used to evaluate the low-temperature performance of asphalt mortar. Additionally, they had a good linear relationship, and the evaluation results were consistent. Meanwhile, T_2_, E_60_, and tan(δ)_max_ indicators can effectively evaluate the high-temperature performance of asphalt mortar. Asphalt plays a key role in the performance of asphalt mortar. Mortars with neat asphalt A70 and modified asphalt AR had the worst and best high- and low-temperature performances, respectively. Furthermore, the finer gradation improved the low-temperature performance of asphalt mortar, while the coarser gradation improved the high-temperature properties of modified asphalt mortars but had the opposite effect on neat asphalt A70.

## 1. Introduction

Asphalt mixtures are widely used in highway construction, and their performance can be studied from various scales including asphalt binder, mastic, mortar (or fine aggregate matrix, FAM), and mixtures [1]. Asphalt mortar is a soft phase filled in the coarse aggregate particles of the asphalt mixture. It is composed of asphalt binder, voids, filler particles, and fine aggregate particles with specific gradation [2,3]. Asphalt mortar significantly impacts the road performance and service life of asphalt mixtures [4,5,6,7]. Therefore, research on the performance of asphalt mortar is useful to further study the material characteristics of asphalt mixtures.

Asphalt mortar is a homogeneous material because its composition is relatively simple. However, some studies have shown that additives, such as composite additives, synthetic fibers, and mineral fillers [8,9,10], can improve the performance of asphalt mortar, and the asphalt, gradation, and asphalt–aggregate ratio are the main factors determining the viscoelastic properties of mortar [11,12]. Researchers hope to gain a better understanding of the road performance of asphalt mixtures by evaluating asphalt mortar, because performance tests on asphalt mortar are more efficient and repeatable [3,13].

In previous research, various test procedures were adopted by different researchers to evaluate the properties of asphalt mortar. Yang et al. [7], Im et al. [14], and Aragão et al. [15] evaluated the low-temperature performance and fracture properties of asphalt mortar usings semicircular bending tests. Meanwhile, Gong et al. [16] used a bending beam rheometer to effectively investigate the low-temperature property of asphalt mortar. Zhang et al. [17] investigated the flexural–tensile rheological behavior of asphalt mortar by the beam bending creep test. The stress relaxation behavior of cement and asphalt mortar under different initial strain levels was investigated by Fu [18]. Zhu et al. [19] evaluated the fatigue performance of fine aggregate matrix containing recycled asphalt shingles using the conventional time sweep test and a proposed strain sweep test. Furthermore, the dynamic mechanical analysis method (DMA) has widely been used recently to analyze the viscoelastic properties of asphalt mortar. Fu et al. [20] evaluated the viscoelastic behavior of cement and asphalt mortar using the DMA method at a temperature range of −40 to 60 °C. Yu et al. [21] evaluated the effect of ultraviolet aging on the dynamic mechanical properties of SBS-modified asphalt mortar using the bending and dynamic moduli. They found that the bending modulus of SBS-modified asphalt mortar increased with the increase in UV intensity and aging time. Additionally, they found that the storage and complex moduli gradually increased with the intensity of aging, but the aging impact of the loss modulus and factor were not obvious. Fang et al. [22] studied the effects of mix parameters on the dynamic mechanical properties of the asphalt mortar using the DMA method. They quantitatively established the relationships between dynamic mechanical properties and the volume fraction of phases in asphalt mortar. In sum, testing and evaluating viscoelastic properties of asphalt mortar using the DMA method is popular because of its simplicity, reproducibility, and flexibility.

The viscoelasticity of asphalt mortar exhibits different phase states over a wide temperature range, including glass brittle solid, elastic rubber, or a viscous fluid, which is directly related to the road performance of asphalt mixture [23]. The DMA method can be used to study the phase transition of viscoelastic materials [24]. The characteristic temperature corresponding to the transition of a material from one phase state to another is called the phase transition temperature, which can be obtained from DMA testing by measuring the complex modulus of the viscoelastic material at different temperatures. When a certain viscoelastic material is subjected to sinusoidal stress, it exhibits delayed strain. The complex modulus, storage modulus, loss modulus, and phase angle tangent value curve can be obtained using the dynamic mechanical test method. The relationship between them is as follows:

(1) $$E^{\prime }=E\cos(\delta )$$

(2) $$E^{\prime \prime }=E\sin(\delta )$$

(3) $$\tan(\delta )=\;\frac{E^{\prime \prime }}{E^{\prime }}$$

where E is the complex modulus; E′ is the storage modulus; E″ is the loss modulus; δ is the phase angle.

The temperature corresponding to the inflection point of the complex modulus curve or the temperature corresponding to the peak point of the loss modulus curve and tangent of the phase angle curve is called the phase transition temperature or glass transition temperature [23,24,25,26]. The phase transition temperature of a viscoelastic material is an important criterion in predicting its field performance. However, although many studies on the viscoelastic properties of asphalt mortar used the DMA method, few evaluations on the phase transition characteristics and phase transition temperature of asphalt mortar exist.

In this study, six asphalt mortars with three asphalt and two gradations were prepared and tested using the DMA method. The phase transition temperature and other characteristic indices of asphalt mortar were obtained through the mechanical response curves, and the high- and low-temperature performance of asphalt mortars were evaluated.

## 2. Materials and Methods

### 2.1. Materials

**Figure 1:**
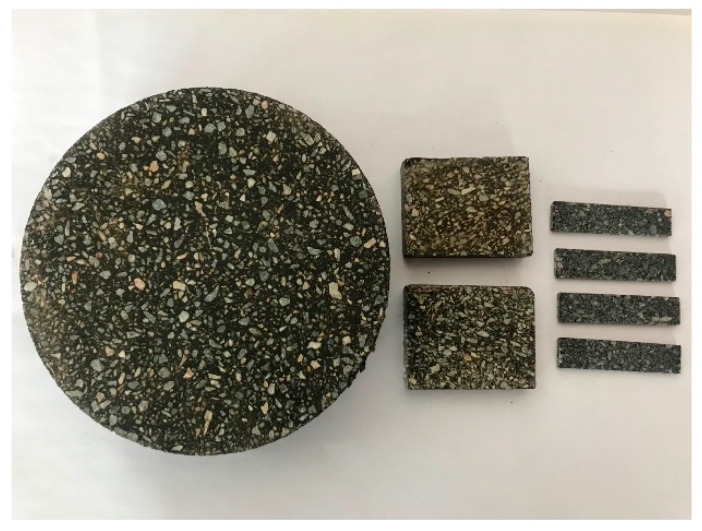
Asphalt mortar samples.

In this study, neat asphalt A70, SBS-modified asphalt, and rubber asphalt AR with 22 wt% crumb-rubber-powder content were used. Table 1 shows the basic technical indices of asphalt. Limestone aggregate and mineral powder were used to make asphalt mortar, and its technical indices met the technical specifications for asphalt pavement construction. Asphalt mortar was formed using two grading methods: G1 and G2. The maximum particle size of the aggregate used was less than or equal to 2.36 mm, and Table 2 shows the specific gradation. The specimens tested in this work were produced by the Superpave gyratory compactor, and the air void level was measured as shown in Table 3. As shown in Figure 1, the cylindrical specimen was cut with a length, width, and height of 60, 13, and 3 mm, respectively.

### 2.2. Methods

**Figure 2:**
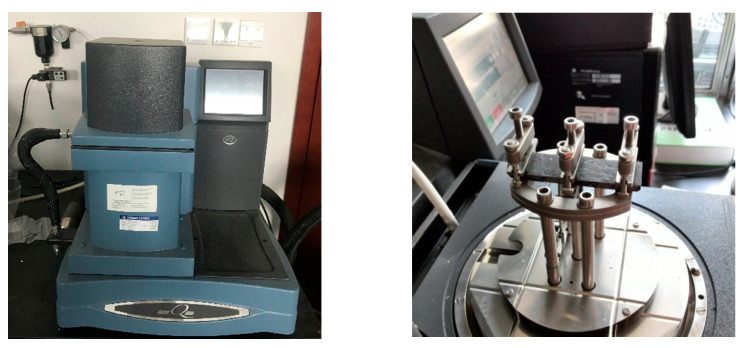
DMA instrument and dual cantilever clamp.

The DMA test was conducted using a TA instrument DMA Q-800 apparatus (New Castle, DE, USA) and dual cantilever loading clamp as shown in Figure 2. The temperature-sweep mode was used with a fixed frequency of 1 Hz and strain of 50 με. The criteria for using a strain of 50 με were based on ensuring that the materials remained in the linear viscoelastic zone and avoiding the influence of noise caused by too small strain on the test accuracy [25]. The test was conducted at a temperature ranging from −35 to 75 °C and a rate of heating of 2 °C/min. Liquid nitrogen was used for temperature control during the test. Each specimen was fixed on the double cantilever clamp with the force of fixed torque. The furnace was turned off, and the temperature was cooled down to −35 °C and held constant for 10 min. Then, the temperature was raised at a heating rate of 2 °C/min. At the same time, the dynamic load was applied to the specimen, and the complex modulus, storage modulus, loss modulus, phase angle, and other information were collected. Two specimens were tested from each asphalt mortar, and the results were averaged.

**Figure 3:**
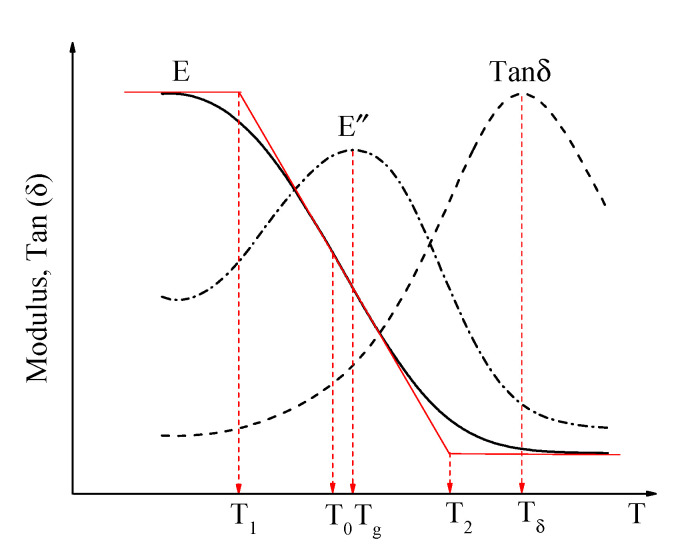
Determination of the glass transition temperature from dynamic testing.

Figure 3 shows the complex modulus, loss modulus, and tan(δ) curves obtained from the temperature-sweep test. The curve of the complex modulus, varying with the temperature, shows an inverse S-shape. In this study, the modulus curve was fitted using the Boltzmann function [24] in the temperature range of −30 to 70 °C as shown in Equation (4).

(4) $$y=\frac{A_{1}-A_{2}}{1+e^{\left(T-x_{0}\right)/\mathrm{dx}}}+A_{2}$$

where $A_{1}$ and $A_{2}$ are the maximum and minimum modulus, respectively; $x_{0}$ and $d_{x}$ are the shape parameters of the modulus curve.

According to the characteristics of the complex modulus curve and the fitting parameters of the Boltzmann function, three-phase transition temperature points can be obtained from the complex modulus curve to describe the phase transition characteristics of asphalt mortar. The three-phase transition temperatures are as follows:

- The temperature corresponding to the midpoint ($x_{0},y_{0}$) of the complex modulus curve is defined as T_0_, T_0_ = ${\;x}_{0}$. This is also the temperature point where the complex modulus decreases the fastest with increasing temperature;
- The temperature corresponding to the intersection of the midpoint tangent of the complex modulus curve and the asymptote in the low-temperature zone is defined as $T_{1}$, $T_{1}=x_{0}-2d_{x}$. T_1_ is the temperature corresponding to the change in asphalt mortar from a glassy to a rubbery state, which can be considered the glass transition temperature of the asphalt mortar;
- The temperature corresponding to the intersection of the midpoint tangent of the complex modulus curve and the asymptote in the high-temperature zone is defined as $T_{2}$, $T_{2}=x_{0}+2d_{x}$. This temperature reflects the phase behavior characteristics of asphalt mortar in the high-temperature zone. The loss modulus and tan(δ) curves have distinct peak points, and the temperatures corresponding to the peak points are denoted as T_g_ and T_δ_, respectively, which can be referred to as the glass transition temperature [23,25].

This study obtained five-phase transition temperatures through the DMA test as shown in Figure 3. The phase transition temperatures were used to evaluate the high- and low-temperature performance of asphalt mortar.

## 3. Results and Discussion

### 3.1. Complex Modulus

**Figure 4:**
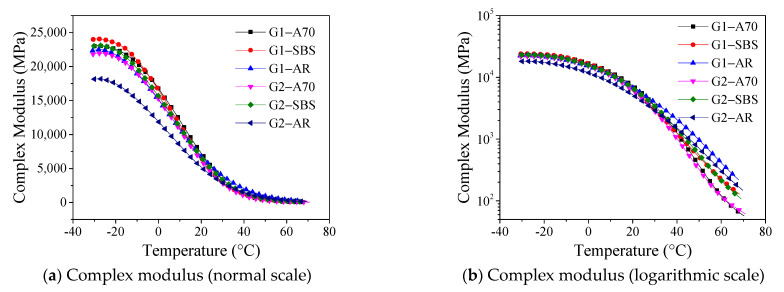
Complex modulus curve (normal and logarithmic scales).

Figure 4 shows the complex modulus for the tested asphalt mortar drawn on normal and logarithmic scales. In the range of temperature less than T_1_, the rate of the modulus increasing with decreasing temperature decreased until the modulus remained constant. At this temperature, the asphalt mortar was close to the elastomer. The complex modulus decreased rapidly with increasing temperature in the temperature range T_1_–T_2_. Although the value of complex modulus was small in the high-temperature region where the temperature was greater than T_2_, it can be seen in the logarithmic scale (Figure 4b) that the change rate of the complex modulus of different types of asphalt mortar showed obvious variability with the increase in temperature. This difference was mainly due to the different types of asphalt and the minor impact of mineral aggregate gradation. The complex modulus of the A70 asphalt mortar was the smallest, and the rate of modulus decrease with increasing temperature was the largest, whereas that of the AR asphalt mortar was the opposite.

**Figure 5:**
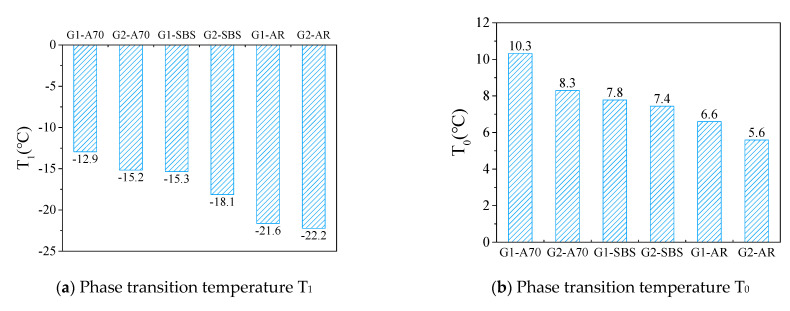

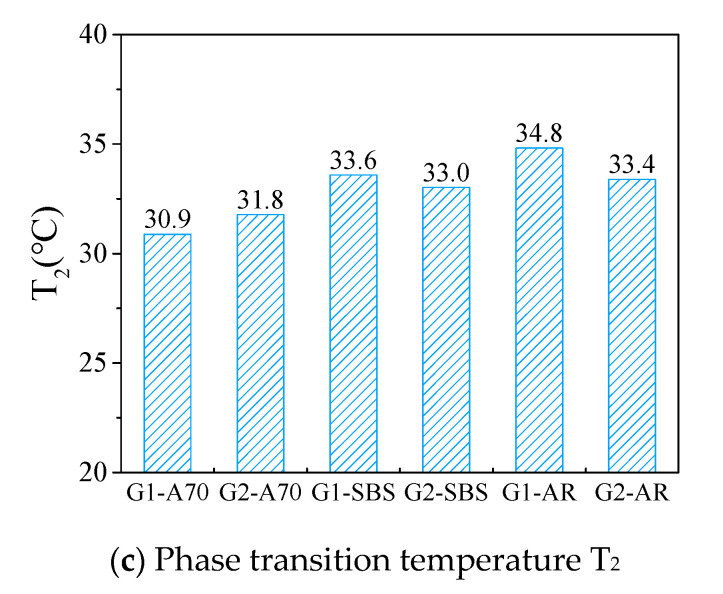
Phase transition temperature obtained from complex modulus (i.e., T_1_, T_0_, and T_2_).

The Boltzmann function model was used to fit the complex modulus curve, and the fitting coefficients *R*^2^ were greater than 0.999. The phase transition temperatures T_0_, T_1_, and T_2_ of the asphalt mortar were calculated according to the fitting parameters. Figure 5a shows the phase transition temperature T_1_ of six kinds of asphalt mortar. The highest temperature T_1_ of G1-A70 was −12.9 °C, and the lowest temperature T_1_ of G2-AR was −22.2 °C. When the aggregate gradation was the same, the phase transition temperature T_1_ of the three samples were A70, SBS, and AR from high to low. Due to the different types of asphalt, the maximum and minimum phase transition temperatures T_1_ of the mortar were nearly 10 °C. The characteristic temperature T_1_ of the asphalt mortar with G1 gradation was higher than G2 when the asphalt was the same. Aggregate gradation G1 was coarser than that of G2, resulting in a difference in asphalt film thickness and porosity, which affects the transition temperature of the mortar. In this study, the transition temperature T_1_ of asphalt mortar with coarse gradation, thick asphalt film, and large porosity was relatively higher. However, the maximum difference in T_1_ caused by different aggregate gradations was less than 3 °C, indicating that asphalt was still the main factor influencing the transition temperature T_1_ of asphalt mortar.

**Figure 6:**
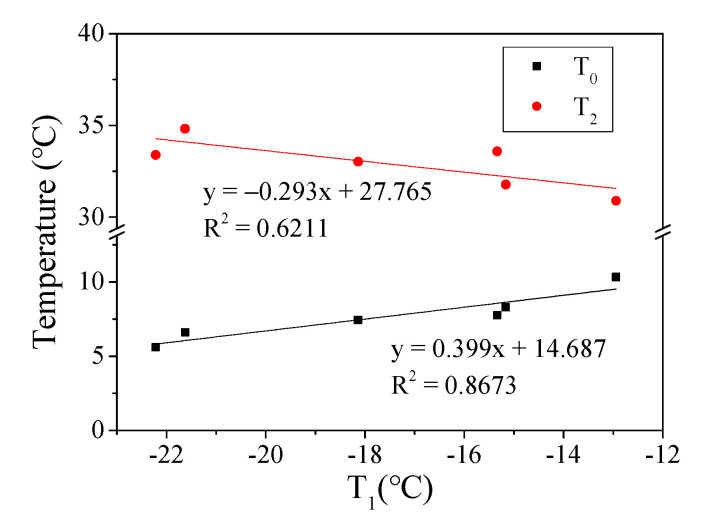
Relationship between T_1_ and T_0_ and T_2_.

Figure 5b shows the phase transition temperature (T_0_) of asphalt mortar where T_0_ and T_1_ had the same change trend. When combined with Figure 6, it is clear that T_0_ and T_1_ had a good linear relationship with a fitting coefficient *R*^2^ greater than 0.86. Due to the fact of this good relationship, it can be deduced that the phase transition temperatures T_1_ and T_0_ can be used to evaluate the low-temperature performance of asphalt mortar. Furthermore, the lower the phase transition temperature, the better the low-temperature crack resistance of asphalt mortar. In this study, the order of the low-temperature performance of the mortar prepared usings three kinds of asphalt was AR > SBS > A70.

Figure 5c shows the phase transition temperature T_2_ of asphalt mortar. Phase transition temperature T_2_ can be considered the characteristic temperature at which asphalt mortar changes from a rubbery to a viscous state. The higher the temperature, the better the high-temperature performance of the mortar. Figure 6 shows that temperature T_2_ had a poor linear correlation with T_1_, with the fitting coefficient *R*^2^ = 0.62. The maximum phase transition temperature T_2_ of G1-AR asphalt mortar was 34.8 °C, and the minimum T_2_ of G1-A70 asphalt mortar was 30.9 °C. The T_2_ of modified asphalt mortar with coarse grading (G1) was relatively high. Meanwhile, the three kinds of asphalt had high-temperature performance in the order AR > SBS > A70. In this study, the phase transition temperature T_2_ of six kinds of asphalt mortars had small variability, indicating that the asphalt type and aggregate gradation have little influence on the characteristic temperature T_2_ of the mortar.

Figure 4b shows that the modulus of asphalt mortar varied significantly in the high-temperature range where the temperature was greater than 40 °C. The index usually evaluates the high-temperature performance of pavement materials under 60 °C. The complex modulus of asphalt mortar at 60 °C was calculated in this study using the parameters of the Boltzmann fitting equation as shown in Table 4. The E_60_ value of mortar with the same asphalt was close, but there was a significant difference when the asphalt was different. The asphalt mortar G1-A70 had the worst performance at a high temperature with the smallest E_60_ value of 33 MPa. The asphalt mortar G1-AR had the best high-temperature performance with the largest E_60_ value of 450 MPa. T_2_ and E_60_ were used as indices to evaluate the high-temperature performance of six mortars, and the results were consistent.

### 3.2. Loss Modulus

**Figure 7:**
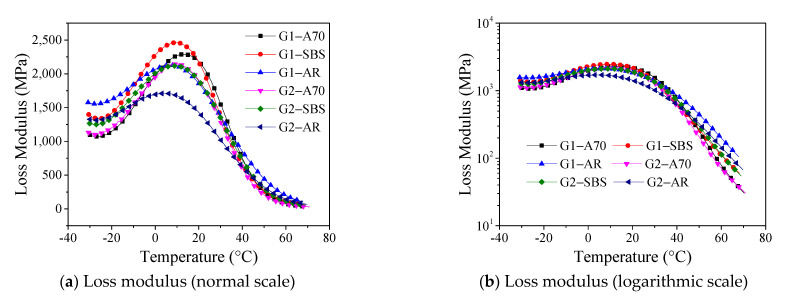
Loss modulus curve (normal and logarithmic scales).

**Figure 8:**
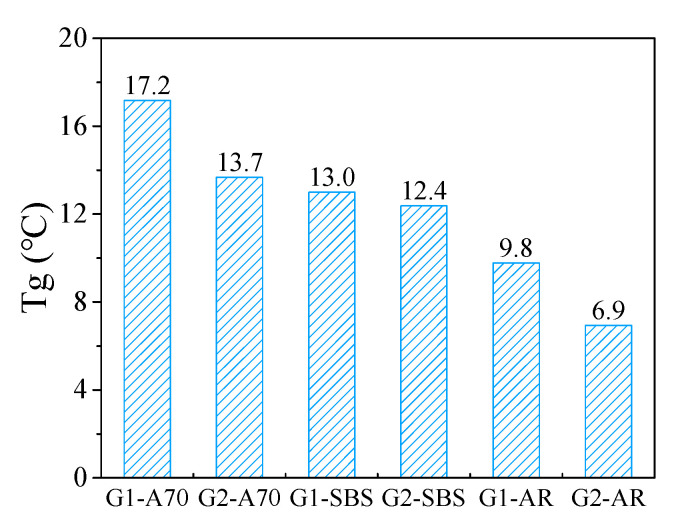
Phase transition temperature T_g_.

The loss modulus is the amount of energy dissipated due to the irreversible viscous deformation when a material deforms under stress, and it reflects the material’s viscosity. The temperature corresponding to the peak point of the loss modulus curve can be used as the glass transition temperature to evaluate the low-temperature properties of materials. Figure 7 shows the loss modulus for the tested asphalt mortar drawn on normal and logarithmic scales. It can be observed from this figure that asphalt type and aggregate gradation cause significant differences in loss modulus across a wide temperature range. Figure 8 shows the phase transition temperature T_g_ of asphalt mortar obtained from the loss modulus temperature curve. The highest phase transition temperature T_g_ of asphalt mortar G1-A70 was 17.2 °C, and the lowest phase transition temperature of G2-AR was 6.9 °C. With the same asphalt, the phase transition temperature T_g_ of mortar with an aggregate gradation of G1 was higher than that of G2.

**Figure 9:**
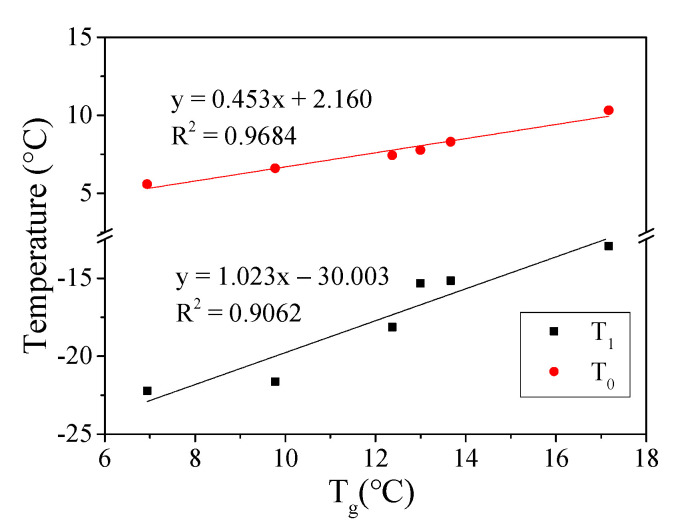
Relationship between T_g_ and T_1_ and T_0_.

The glass transition temperature is an important parameter to evaluate the low-temperature performance of materials. T_1_, T_0_, and T_g_ obtained using the DMA method can be used as glass transition temperatures to evaluate the low-temperature performance of asphalt mortar. Figure 6 and Figure 9 show a good linear relationship between T_1_, T_0_, and T_g_, and the evaluation results of the low-temperature performance of six kinds of asphalt mortar were consistent.

### 3.3. Tan(δ) (E″/E′)

**Figure 10:**
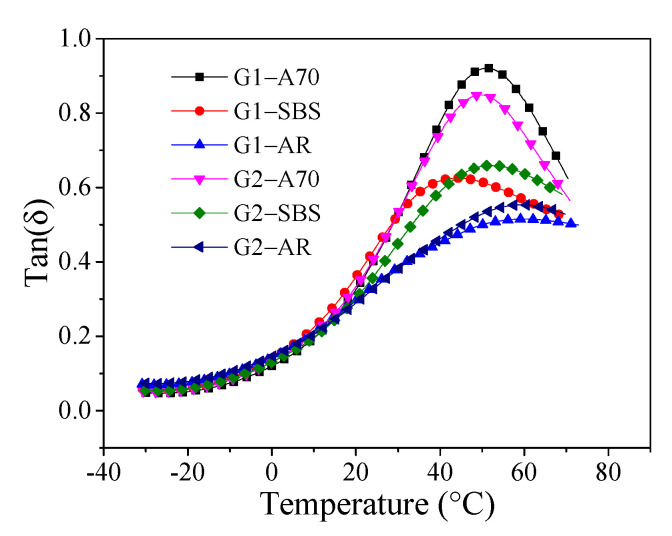
The curve of tan(δ).

Figure 10 shows the tan(δ) curve of asphalt mortar. In the test temperature range, the tan(δ) value of the asphalt mortar first increased and then decreased. The tan(δ) value gradually increased as the temperature rose, and the rate of increase was slow in the low-temperature range (less than 0 °C). When the temperature exceeds 20 °C, the tan(δ) value rapidly increased and decreased after reaching its maximum. In the low-temperature region, the tan(δ) values of the six kinds of asphalt mortars had little difference. Still, in the temperature range of 20–70 °C, the tan(δ) temperature curves of the different kinds of asphalt mortar varied significantly. When the temperature exceeded 20 °C, the tan(δ) value and its increasing rate of A70 asphalt mortar were the largest, while the AR asphalt mortar was the opposite. The tan(δ) curves of the two aggregate gradation mortars were similar when the asphalt was the same, which indicates that the asphalt type was the main factor affecting the tan(δ) curve of the asphalt the mortar.

Tan(δ) is the ratio of loss modulus to storage modulus. The larger the tan(δ) value, the larger the proportion of loss modulus, the greater the irreversible deformation capacity of the material under the action of external force, and the closer the material is to the viscous phase. In the high-temperature region, the higher the tan(δ) value of asphalt mortar, the more likely the irreversible viscous deformation of asphalt mortar will occur when the asphalt mortar is subjected to external force, indicating that the high-temperature deformation resistance of asphalt mortar is worse. Meanwhile, the lower the peak temperature of the tan(δ) value curve, the worse the high-temperature performance of mortar.

**Figure 11:**
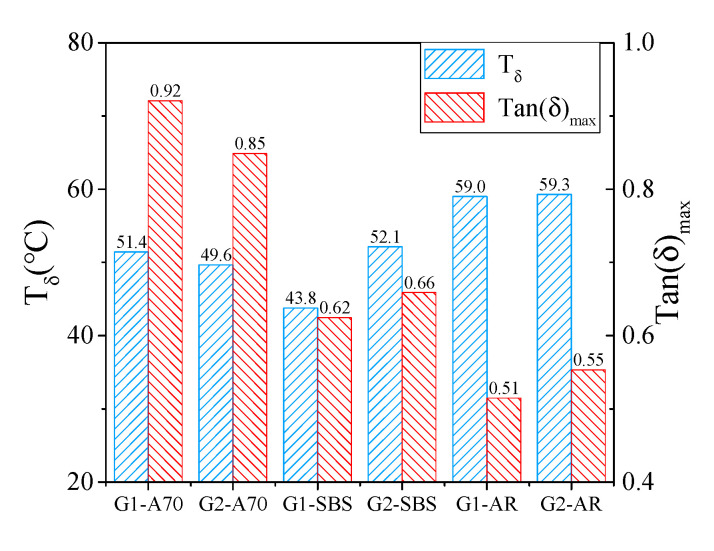
Maximum tan(δ) and corresponding temperature T_δ_.

Figure 11 shows the maximum tan(δ) value and the corresponding temperature T_δ_. Asphalt mortar G1-SBS had the lowest phase transition temperature T_δ_ of 43.8 °C, whereas asphalt mortar G2-AR had the highest at 59.3 °C. This result was inconsistent with the evaluation results of indicators T_2_ and E_60_. This is because asphalt mortar is not a uniformly simple material composition, and its phase transformation characteristics under a high-temperature range are more complex. However, asphalt mortar G1-A70 had the largest Tan(δ)_max_ value of 0.92, and asphalt mortar G1-AR had the smallest Tan(δ)_max_ value of 0.51. The Tan(δ)_max_ values of two mortars with the same asphalt were similar, and the order of the high-temperature performance of the asphalt mortar prepared with the same aggregate gradation of three kinds of asphalt was AR > SBS > A70. This was different from the evaluation result of phase transition temperature T_δ_ but consistent with the evaluation results of indicators T_2_ and E_60_, and it was also consistent with the high-temperature grade (PG grade) of asphalt as shown in Table 1. Therefore, Tan(δ)_max_ can more effectively evaluate the high-temperature performance of asphalt mortar.

Asphalt plays a key role in the high- and low-temperature performance of asphalt mortar. The mortars formed by asphalt AR and SBS had the better high- and low-temperature performance. This was mainly because polymer SBS and crumb rubber powder absorbed the light component of neat asphalt, resulting in swelling and changing the phase characteristics of asphalt. The interactions between modifier and asphalt were strengthened, leading to the improved high-temperature performance and toughness. The swelled SBS and crumb rubber particles made the asphalt more elastic and flexible; therefore, the elasticity and low-temperature performance of the asphalt were also improved.

## 4. Conclusions

In this study, three kinds of asphalt and two kinds of aggregate gradation were used to form six kinds of asphalt mortar samples. The viscoelastic properties of six kinds of asphalt mortars were tested using the DMA method. The temperature-sweep of asphalt mortar was conducted with a dual cantilever clamp under the conditions of a fixed strain level, frequency, and heating rate. The dynamic response curves of asphalt mortar under flexural and tensile modes were obtained including complex modulus, storage modulus, loss modulus, and tan(δ).

The Boltzmann function model was used to fit the complex modulus curve, and the phase transition temperatures T_1_, T_0_, and T_2_ of asphalt mortar were obtained according to the fitting parameters. The loss modulus and tan(δ) curves had clear peak points, and the phase transition temperatures T_g_ and T_δ_ corresponding to peak points were obtained. Meanwhile, the complex modulus at 60 °C (E_60_) and the maximum tan(δ) (Tan(δ)_max_) were used as indicators to evaluate the high-temperature performance of asphalt mortar. The following conclusions were drawn:

a) The phase transition temperatures T_1_, T_0_, and T_g_ can be used as the glass transition temperature of asphalt mortar, and they had a good linear relationship. T_1_, T_0_, and T_g_ effectively evaluated the low-temperature performance of asphalt mortar, and the evaluation results of the three indices were consistent. Mortars with neat asphalt A70 had a significantly higher T_1_, T_0_, and T_g_ and poor low-temperature performance, while mortars with modified asphalt AR had the best low-temperature performance. The finer gradation had a positive effect on the low-temperature performance of asphalt mortar.
b) Phase transition temperature T_2_, complex modulus at 60 °C (E_60_), and the maximum tan(δ) (Tan(δ)_max_) can be used to evaluate the high-temperature performance of asphalt mortar. The mortar formed by asphalt AR had the best high-temperature performance, followed by asphalt SBS and A70. Coarser gradation had a good effect on the high-temperature performance of the modified asphalt, but it had the opposite effect on the neat asphalt A70.
c) Asphalt plays a key role in the high- and low-temperature performance of asphalt mortar. In this study, the order of high- and low-temperature performance was AR > SBS > A70.

The temperature-sweep test based on the DMA method can effectively evaluate the phase transition characteristics of asphalt mortar in a wide temperature range, and the significance of the evaluation indicators are clear. The reliability, effectiveness, and practicability of the method for evaluating the high- and low-temperature performance of asphalt mortar using phase transition temperature will be further validated in future research by combining with the road performance research of asphalt mixture.

## Figures and Tables

**Table 1:** Basic technical indices of asphalt.

Asphalt	Penetration (25 °C)/0.1 mm	Softening Point/°C	Ductility (5 cm/min, 10 °C)	PG Grade
A70	67	49.0	55	PG64-22
SBS	63	72.7	49	PG82-22
AR	39	72.6	-	PG88-28

**Table 2:** Gradation of aggregates used in the study.

Percent Passing /%wt	Sieve Size/mm						
	4.75	2.36	1.18	0.6	0.3	0.15	0.075
G1	100	67.8	46.2	31.7	21.6	14.7	10
G2	100	84.8	69.8	55.1	40.1	25	10

**Table 3:** Scheme of asphalt mortar molding.

Asphalt Mortar	Asphalt	Gradation	Asphalt–Aggregate Ratio/%	Porosity/%
G1-A70	A70	G1	6.5	3.6
G1-SBS	SBS	G1	6.5	3.7
G1-AR	AR	G1	6.5	3.4
G2-A70	A70	G2	6.5	2.8
G2-SBS	SBS	G2	6.5	2.8
G2-AR	AR	G2	6.5	2.7

**Table 4:** The complex modulus at 60 °C.

Asphalt Mortar	E_60_/MPa
G1-A70	33
G2-A70	41
G1-SBS	157
G2-SBS	150
G1-AR	450
G2-AR	330

## Data Availability

Not applicable.
